# The Roles of Heat Shock Protein-60 and 70 and Inflammation in Obesity-Related Kidney Disease

**DOI:** 10.7759/cureus.28675

**Published:** 2022-09-01

**Authors:** Özden Yıldırım, Erhan Tatar

**Affiliations:** 1 Internal Medicine Department, University of Health Sciences Bozyaka Research and Training Hospital, İzmir, TUR; 2 Nephrology Department, University of Health Sciences Bozyaka Research and Training Hospital, İzmir, TUR

**Keywords:** inflamation, heat shock protein-70, heat shock protein-60, kidney disease, obesity

## Abstract

Introduction

The exact mechanisms of obesity-related kidney disease (ORKD) are not fully known. Heat shock proteins (HSPs) may play a role in ORKD mechanisms because of their role in cell apoptosis, cytoprotection, and inflammatory processes. We aimed to determine the role of circulating serum HSP-60 and HSP-70 levels as a biomarker for ORKD.

Materials and methods

This study included 40 ORKD patients, 40 obese age-matched and sex-matched controls with similar body mass index (BMI), and 40 healthy controls. Their serum biochemical and hemogram parameters as well as HSP-60 and HSP-70 levels were evaluated and compared. Their neutrophil-to-lymphocyte ratio (NLR) and C-reactive protein levels were assessed to define inflammation.

Results

The patients had significantly higher HSP-60 levels than the obese and healthy controls (537.58 ± 170.35, 430.80 ± 110.61, and 371.85 ± 76.34, respectively; p<0.00). The results revealed that the 24-hour urinary protein levels had a positive correlation (r= 0.544), whereas the glomerular filtration rate had a negative correlation (r = 0.38) with the serum HSP-60 level. According to the regression analysis performed on the HSP-60 and 24-hour urinary protein excretion levels, an increase in the HSP-60 level significantly increased the 24-hour urinary protein excretion rate (r=0.15; p<0.005). The HSP-60 levels were correlated with inflammatory markers

Conclusion

The serum HSP-60 levels increased in patients with ORKD. This increase was correlated with 24-hour urinary protein excretion. Increased circulating levels of HSP-60 may play a role in the initiation and/or progression of renal damage and inflammation. HSP-60 is a potential biomarker for ORKD. However, additional information and studies are required to further elucidate this finding.

## Introduction

Obesity is a major health problem, and its prevalence is increasing along with the global obesity epidemic, which is affected by genetic, physiological, and sociocultural factors. Obesity is a critical, correctable, and independent risk factor for the development of chronic kidney disease (CKD). A positive correlation exists between increased body mass index (BMI) and proteinuria even before CKD progression [1]. Furthermore, a high BMI is associated with a low estimated glomerular filtration rate (eGFR), rapid e-GFR loss over time, and a high incidence of end-stage renal disease (ESRD) [2]. 

Heat shock protein (HSP) molecules have different roles depending on their intracellular or extracellular expression. They are mainly involved in the degradation and clearance of misfolded proteins as well as in chaperone-mediated autophagy, protein quality control mechanisms, cell signaling, stress response formation, and kidney self-defense systems [3]. In rat models, HSP-60 stains primarily in the proximal tubule cells and less intensely in the distal convoluted tubule cells. In addition, HSP-60 is detected in the thick ascending limbs of Henle’s loop [4]. HSP-73 is the main protein of the HSP-70 family that is expressed in normal renal tissue [4,5]. HSP-73 is specifically expressed in the Bowman’s capsule, whereas HSP-72 is expressed in the renal cortex and medulla, also, there is an increase in inducible HSP-72 expression in the kidney after exposure to ischemia or toxic damage [6,7]. Extracellular HSPs play a role in inflammation and immunity, but their functions are not entirely clear [8]. They are involved in capturing and presenting intracellular antigens to antigen-presenting cells, and they can also activate T-reg cells and inhibit immunity and inflammation. The pro-immune function hypothesis for extracellular HSPs has mainly been evaluated in studies on the use of molecular chaperone vaccines for cancer therapy [8]. It has been suggested in the literature that HSP molecules may serve as biomarkers for identifying kidney damage [6]. 

Hypertrophy, hyperplasia, and inflammation in adipocytes cause many changes in adipose tissue structures and adipokine secretion. Increased leptin levels in obese patients activate the sympathetic system and cause sodium retention and hypertension. Furthermore, increased leptin levels accelerate kidney disease progression by increasing endothelial dysfunction. The release of proinflammatory cytokines such as interleukin (IL)-6 and tumor necrosis factor-alpha (TNF-α) in the adipose tissue of obese patients causes inflammation and oxidative stress [9,10]. A decrease in the adiponectin levels of obese patients also contributes to renal damage by downregulating protective mechanisms [11].

Although it is known that proinflammatory mediators are secreted from adipocytes, the physiological signals that trigger their release have not been fully determined. HSP-60 is an important inductor in the release of proinflammatory cytokines from the adipose tissue [12]. In addition, HSP-60 levels have been demonstrated to be elevated in obese patients, and this elevation correlates with inflammatory markers [13].

HSP-60 has recently been identified as a new molecular link in obesity-related inflammation and insulin resistance [14]. The role of HSP molecules in obesity-related kidney disease (ORKD) and the relationship between inflammatory markers have not been studied yet. We aimed to evaluate the role of HSPs and their relationship with inflammation and proteinuria in specific patient groups.

## Materials and methods

Study design

This study was designed as a cross-sectional observational study. It included 120 participants who were consecutively admitted to our internal medicine and nephrology outpatient clinics. 

This study comprised 40 obese CKD patients who did not have diabetes and 40 obese controls who were of similar age, sex, mean BMI, and normal renal function, and 40 healthy controls. We define ORKD as the presence of proteinuria above 300 mg/day or GFR below 60 mL/min, as described in the literature [9].

The inclusion criteria for the study were individuals between 18 and 60 years old who had a BMI of over 30 without diabetes mellitus. The weight (kg)/height (m2) formula was used to calculate the BMI. The main exclusion criteria were the presence of comorbidities (such as diabetes, polycystic kidney disease, and nephrocalcinosis) that may cause chronic renal disease. Demographic data and patient history were recorded.

Ethical consideration 

An informed consent form was signed by the participants in this study. This study was approved by the Ethics Committee of the University of Health Sciences Bozyaka Training and Research Hospital, Turkey, on June 30, 2020, with decision number 248.

Laboratory analysis

The hemogram and biochemical parameters of all the participants, including urea, creatinine, uric acid, and C-reactive proteins (CRPs), were studied. Detailed analyses of the biochemical parameters and a complete blood count (CBC) were performed on all the participants. The neutrophil count was divided by the lymphocyte count, and the neutrophil-to-lymphocyte ratio (NLR) was calculated. The e-GFR was calculated using the Modification of Diet in Renal Disease formula. The biochemical parameters were analyzed using an Olympus AU2700 Chemistry analyzer (Beckman Coulter, Brea, CA). Urine samples were collected over 24 hours for proteinuria analysis. Each participant’s blood was collected in a clot-activating tube with a gel separator, centrifuged at 1500 × g for 10 min, and then stored at -80 °C until HSP analysis. The serum HSP-60 and HSP-70 levels were determined using a ready kit and the sandwich enzyme-linked immunosorbent assay (ELISA) method (SinoGeneClon Biotech Co. Ltd). Analyses were made based on the instructions written in the kit prospectuses. An ELISA device (Thermo Scientific Multiskan GO model, Thermo Scientific Corp., Waltham, MA) with a 450 nm wavelength was used for spectrophotometric measurements. The serum HSP-60 and HSP-70 concentrations were calculated by creating an absorbance concentration graph. The results are expressed as ng/ml. 

Statistical method

SPSS Statistics v. 18.0 (IBM Corp., Armonk, NY) was used for statistical analysis. The data is presented as mean ± SD. In this study, a one-way analysis of variance (ANOVA) was performed on the three groups. A homogeneity of variance test was performed for all the parameters. Bonferroni and Tamhane’s tests were used for post hoc analysis. Since the three groups were compared, the new p-value was determined as 0.017 by applying Bonferroni correction at the significance level. The relationship between HSP-60, HSP-70, and other demographic and laboratory data was evaluated. 

## Results

Our study included 40 (20 female) ORKD patients, 40 (20 female) obese patients with normal renal function, and 40 (20 female) healthy volunteers. There was no statistically significant difference in terms of age and sex (p=1). Table 1 presents the demographic data of the patients and control groups.

**Table 1:** Demographic data of the patients and control groups. ORKD: Obesity-related kidney disease group. M: male, F: Female, n: number

Group	Age (Mean)	Sex
ORKD group (n=40)	51.74 (6.91)	20M/20F
Obese control group (n=40)	49.88 (8.32)	20M/20F
Healthy control group (n=40)	48.85 (8.63)	20M/20F
P-value	0.41	1

The serum HSP-60, serum HSP-70, BMI, 24-h urinary protein excretion, urea, fasting blood glucose, creatinine, e-GFR, CRP, and NLR levels of the three groups were compared. As expected, there were no differences in the blood glucose levels because the study was conducted on non-diabetic subjects (p=1). The obese patients and obese control group had a statistically significantly higher BMI than the healthy controls (p<0.001). The obese patients had statistically significantly higher levels of GFR, urea, creatinine, 24-h urinary protein excretion, CRP, and NLR than those in the control groups. The HSP-60 levels were relatively high in the patient group (p<0.001), but the HSP-70 levels did not exhibit any significance. Table 2 presents the laboratory results of the participants.

**Table 2:** Laboratory results of patients and controls. HSP: heat shock protein; BMI: body mass index; GFR: glomerular filtration rate; CRP: C-reactive protein; NLR: neutrophil-lymphocyte ratio Group 1 contained obesity-related kidney disease patients, group 2 contained obese controls, and Group 3 contained healthy controls. The mean difference is significant at the 0.017 level.

	Group	Mean±Standard deviation	95% Confidence Interval for Mean		Significance (p-value)
			Lower Bound	Upper Bound	
HSP-60 (ng/mL)	1	537.58±170.35	482.36	592.81	Group 1-2; p=0.01
	2	430.80±110.61	386.12	475.48	Group 1-3; p<0.001
	3	371.85±76.34	327.77	415.93	Group 2-3; p=0.62
HSP-70 (ng/mL)	1	72.61±36.95	60.63	84.59	Group 1-2; p=1
	2	63.95±22.03	55.05	72.84	Group 1-3; p=0.121-3
	3	49.32±52.23	19.16	79.48	Group 2-3; p=0.68
BMI(kg/m^2^)	1	35.2±3.11	34.19	36.20	Group 1-2; p=1
	2	35.05±3.73	33.54	36.55	Group1-3; p=1
	3	26.84±1.64	25.89	27.79	Group 2-3; p=1
24 h urinary protein(g/day)	1	654.31±245.16	314.51	994.12	Group 1-2; p=0.016
	2	108.92±181.27	35.70	182.14	Group1-3; p=0.03
	3	50.35±9.89	44.64	56.07	Group 2-3; p=1
Urea(mg/dL)	1	55.69±35.53	44.17	67.21	Group 1-2; p<0.001
	2	27.69±5.99	25.27	30.11	Group 1-3; p=0.003
	3	27.61±5.36	24.37	30.85	Group 2-3; p=1
Blood glucose(mg/dL	1	94.33±12.82	90.17	98.49	Group 1-2; p=1
	2	92.00±8.70	88.48	95.51	Group 1-3; p=1
	3	95.23±8.74	89.94	100.51	Group 2-3; p=1
Creatinin(mg/dL	1	1.70±0.58	1.51	1.89	Group 1-2; p<0.001
	2	0.89±0.13	0.84	0.94	Group 1-3; p<0.001
	3	0.94±0.17	0.84	1.03	Group 2-3; p=1
Gfr (ml/min/1.73m2)	1	43.09±17.0	37.55	48.63	Group 1-2 p<0.001
	2	90.92±14.79	84.94	96.90	Group1-3; p<0.001
	3	84.35±13.72	76.43	92.27	Group 2-3; p=1
Crp	1	10.55±13.28	6.24	14.86	Group 1-2; p=0.01
	2	3.91±2.64	2.84	9.97	Group1-3 p <0.001
	3	2.61±1.19	1.89	3.33	Group 2-3; p=0.12
NLR	1	1.13±0.18	2.01	2.74	Group 1-2; p<0.001
	2	0.49±0.97	1.45	1.86	Group 1-3; p=0.09
	3	0.63±0.17	1.43	2.20	Group 2-3; p=0.25

We performed post hoc analyses to determine the biochemical and hemogram differences between the patient and control groups. Tamhane’s post hoc multiple comparison test revealed a statistically significant difference in HSP-60 levels between the patient and two control groups (Table 3). This difference was more statistically significant between the healthy control and patient groups (p=0.00).

**Table 3:** Differences in HSP-60 levels between the patient and control groups. The mean difference was significant at 0.017. Group 1 contained obesity-related kidney disease patients, Group 2 contained obese controls, and Group 3 contained healthy controls.

Dependent Variable=HSP-60 Tamhane’s Test	Groups	Mean Difference	Std. Error	p-Value	Lower Bound	Upper Bound
Group 1	2	106.78	34.85	0.010	7.01	206.55
	3	165.73	34.06	0.000	67.15	264.30
Group 2	1	106.78	34.85	0.010	−206.55	−7.01
	3	58.95	29.78	0.158	−28.69	146.59

A regression analysis was performed to compare the HSP-60 and 24-hour urinary protein excretion levels. This analysis revealed that the 24-hour urinary protein excretion level significantly increased as the HSP-60 levels increased (r=0.15; p<0.005). We performed an analysis to determine the correlation between the HSP-60 24-hour urinary protein excretion and e-GFR levels. There was a strong positive correlation between the HSP-60 and urinary protein excretion levels (r=0.539; p<0.005) and CRP levels (r=0.33; p<0.001). In addition, there was a relatively positive correlation between HSP-60 levels and NlLR (r=0.16; p<0.075). There was a strong negative correlation between the e-GFR and HSP-60 levels (r=-0.383; p<0.001).

## Discussion

In this study, we investigated the role of serum HSP-60 and HSP-70 levels in nondiabetic ORKD patients. We included both obese and healthy controls to assess the significance of the study. The patient group had significantly higher serum HSP-60 levels than the control group. This increase was more significant between the healthy control and patient groups (p<0.001). Conversely, there was no significant difference between the HSP-70 levels of the three groups.

Previous studies have investigated the relationship between HSPs and obesity and metabolic syndrome. It has been stated that intracellular HSP-70 and HSP-27 expression may play an important role in the development of metabolic syndrome in obese individuals [15]. The literature has demonstrated that intracellular HSP-70 expression is decreased in the muscle tissue of obese patients and that serum and liver HSP-70 levels are low in diabetic monkeys [16,17]. Although it has been suggested that intracellular HSP-70 molecules have a protective effect on insulin resistance and hyperinsulinemia, the effects of extracellular HSP-70 molecules on obesity are not yet fully known [18]. Sell et al. reported that HSP-60 levels were high in morbidly obese patients but that they decreased after bariatric surgery, and this decrease had a correlation with inflammatory markers and cardiovascular disease risk [13].

HSP molecules are one of the basic body defense mechanisms that are active during stress. However, there are limited studies on HSP molecules in renal diseases. It has been demonstrated that HSP-70 levels are elevated in chronic glomerulonephritis and that HSP-60 mRNA and protein levels are elevated in proximal tubular cells after heat stress [19,20]. A previous study demonstrated increased HSP-25, HSP-60, and HSP-72 expression levels in the renal outer medulla of a diabetic animal model [21]. A study conducted in Egypt demonstrated that HSP-60 may be a biomarker for renal failure that develops after septic shock in children [22]. HSP molecules have been suggested as biomarkers and prognostic factors for CKD [19,23,24]. Similarly, we also identified a positive correlation between HSP-60 levels and 24h proteinuria. A study on the role of heat shock in atherosclerosis suggested that HSP-70 plays a role in cytoprotection, whereas HSP-60 acts as an autoantigen and triggers an immune response [25].

Hauffe et al. demonstrated that decreased HSP-60 levels in mice result in beneficial changes in adipose tissue morphology, body weight, and insulin resistance [26]. We came to a similar conclusion in this study because of the elevated HSP-60 levels in the patient group and the relationship between this elevation and proteinuria and inflammatory markers. We believe that HSP-60 levels are closely associated with renal disease in obese patients, but the role of extracellular HSP-70 in obesity is not fully understood.

In this study, the obese patient group had statistically significantly high CRP and NLR levels. This may be due to the high inflammatory state caused by obesity and CKD. In recent years, researchers have discussed the role of inflammation in obesity, revealing that obesity is a subclinical inflammatory disease. IL-6 and IL-8, transforming growth factor-β, fibroblast growth factor, epidermal growth factor, TNF-α, and CRP are some of the factors that are secreted from the adipose tissue [27]. Habich and Sell reported that impaired intracellular stress in adipose tissue is characterized by a deranged heat shock response and defense system in obesity, and they determined how elevated HSP-60 levels in the adipose tissue contribute to inflammation and metabolic disturbances [14]. Märker et al. stated that HSP-60 induces the secretion of proinflammatory mediators from murine adipocytes and may be an important factor in the development of obesity-related metabolic diseases [28].

The limitations of the study were that the study was cross-sectional and we cannot be completely sure if high HSP-60 levels in obesity caused CKD, or if renal failure was diagnosed based on laboratory findings (GFR and 24-hour urinary protein excretion levels), and renal biopsies were not performed on the patients because there was no indication. We believe that our study can be supported with tissue biopsies from obese renal failure animal models that have had their HSP molecules stained.

## Conclusions

This is the first study to focus on HSP molecules and inflammation in nondiabetic obese renal failure patients. This study demonstrated that HSP-60 and inflammatory markers are statistically significantly high in ORKD patients, whereas HSP-70 exhibits no difference. There was a strong positive correlation between HSP-60 and urinary protein excretion. These findings suggest that HSP-60 plays a role in the initiation and/or progression of renal damage and that it is a chaperone that triggers inflammation in obese individuals. The findings of this study will benefit from large multicenter studies and animal models so that their renal biopsies can be evaluated.

## References

[1] Pinto-Sietsma SJ, Navis G, Janssen WM, de Zeeuw D, Gans RO, de Jong PE (2003). A central body fat distribution is related to renal function impairment, even in lean subjects. Am J Kidney Dis.

[2] Foster MC, Hwang SJ, Larson MG (2008). Overweight, obesity, and the development of stage 3 CKD: the Framingham Heart Study. Am J Kidney Dis.

[3] Akan OY, Ortan P, Hosgorler F (2019). Role of HSP in the treatment of internal diseases. Heat Shock Proteins in Neuroscience.

[4] Chebotareva N, Bobkova I, Shilov E (2017). Heat shock proteins and kidney disease: perspectives of HSP therapy. Cell Stress Chaperones.

[5] Beck FX, Neuhofer W, Müller E (2000). Molecular chaperones in the kidney: distribution, putative roles, and regulation. Am J Physiol Renal Physiol.

[6] Komatsuda A, Wakui H, Imai H, Nakamoto Y, Miura AB, Itoh H, Tashima Y (1992). Renal localization of the constitutive 73-kDa heat-shock protein in normal and PAN rats. Kidney Int.

[7] Harrison EM, Sharpe E, Bellamy CO (2008). Heat shock protein 90-binding agents protect renal cells from oxidative stress and reduce kidney ischemia-reperfusion injury. Am J Physiol Renal Physiol.

[8] Srivastava P (2002). Roles of heat-shock proteins in innate and adaptive immunity. Nat Rev Immunol.

[9] Mathew AV, Okada S, Sharma K (2011). Obesity related kidney disease. Curr Diabetes Rev.

[10] Hall JE, Mouton AJ, da Silva AA, Omoto AC, Wang Z, Li X, do Carmo JM (2021). Obesity, kidney dysfunction, and inflammation: interactions in hypertension. Cardiovasc Res.

[11] Dagogo-Jack S, Fanelli C, Paramore D, Brothers J, Landt M (1996). Plasma leptin and insulin relationships in obese and nonobese humans. Diabetes.

[12] Tiss A, Khadir A, Abubaker J (2014). Immunohistochemical profiling of the heat shock response in obese non-diabetic subjects revealed impaired expression of heat shock proteins in the adipose tissue. Lipids Health Dis.

[13] Sell H, Poitou C, Habich C, Bouillot JL, Eckel J, Clément K (2017). Heat shock protein 60 in obesity: effect of bariatric surgery and its relation to inflammation and cardiovascular risk. Obesity (Silver Spring).

[14] Habich C, Sell H (2015). Heat shock proteins in obesity: links to cardiovascular disease. Horm Mol Biol Clin Investig.

[15] Sabbah NA, Rezk NA, Saad MS (2019). Relationship between heat shock protein expression and obesity with and without metabolic syndrome. Genet Test Mol Biomarkers.

[16] Henstridge DC, Whitham M, Febbraio MA (2014). Chaperoning to the metabolic party: The emerging therapeutic role of heat-shock proteins in obesity and type 2 diabetes. Mol Metab.

[17] Bruce CR, Carey AL, Hawley JA, Febbraio MA (2003). Intramuscular heat shock protein 72 and heme oxygenase-1 mRNA are reduced in patients with type 2 diabetes: evidence that insulin resistance is associated with a disturbed antioxidant defense mechanism. Diabetes.

[18] Moura CS, Lollo PC, Morato PN, Amaya-Farfan J (2018). Dietary nutrients and bioactive substances modulate heat shock protein (HSP) expression: a review. Nutrients.

[19] Bielecka-Dabrowa A, Barylski M, Mikhailidis DP, Rysz J, Banach M (2009). HSP 70 and atherosclerosis--protector or activator?. Expert Opin Ther Targets.

[20] Amaral Pedroso L, Nobre V, Dias Carneiro de Almeida C, da Silva Praxedes MF, Sernizon Guimarães N, Simões E Silva AC, Parreiras Martins MA (2020). Acute kidney injury biomarkers in the critically ill. Clin Chim Acta.

[21] Chebotareva NV, Neprintseva NV, Bobkova IN, Kozlovskaia LV (2014). Investigation of 70-kDa heat shock protein in the serum and urine of patients with chronic glomerulonephritis [article in Russian]. Ter Arkh.

[22] Somji S, Todd JH, Sens MA, Garrett SH, Sens DA (2000). Expression of heat shock protein 60 in human proximal tubule cells exposed to heat, sodium arsenite and CdCl(2). Toxicol Lett.

[23] Barutta F, Pinach S, Giunti S (2008). Heat shock protein expression in diabetic nephropathy. Am J Physiol Renal Physiol.

[24] El-Gamasy MA, El-Sadek AE, Fakhreldin AR, Kamel A, Elbehery EG (2018). Heat shock protein 60 as a biomarker for acute kidney injury secondary to septic shock in pediatric patients, Egyptian multicenter experience. Saudi J Kidney Dis Transpl.

[25] Musial K, Szprynger K, Szczepańska M, Zwolińska D (2010). The heat shock protein profile in children with chronic kidney disease. Perit Dial Int.

[26] Hauffe R, Rath M, Schell M (2021). HSP60 reduction protects against diet-induced obesity by modulating energy metabolism in adipose tissue. Mol Metab.

[27] Ellulu MS, Patimah I, Khaza'ai H, Rahmat A, Abed Y (2017). Obesity and inflammation: the linking mechanism and the complications. Arch Med Sci.

[28] Märker T, Sell H, Zillessen P (2012). Heat shock protein 60 as a mediator of adipose tissue inflammation and insulin resistance. Diabetes.

